# Efficient Constraint Handling in Electromagnetism-Like Algorithm for Traveling Salesman Problem with Time Windows

**DOI:** 10.1155/2014/871242

**Published:** 2014-02-27

**Authors:** Alkın Yurtkuran, Erdal Emel

**Affiliations:** Department of Industrial Engineering, Uludag University, Görükle Campus, 16059 Bursa, Turkey

## Abstract

The traveling salesman problem with time windows (TSPTW) is a variant of the traveling salesman problem in which each customer should be visited within a given time window. In this paper, we propose an electromagnetism-like algorithm (EMA) that uses a new constraint handling technique to minimize the travel cost in TSPTW problems. The EMA utilizes the attraction-repulsion mechanism between charged particles in a multidimensional space for global optimization. This paper investigates the problem-specific constraint handling capability of the EMA framework using a new variable bounding strategy, in which real-coded particle's boundary constraints associated with the corresponding time windows of customers, is introduced and combined with the penalty approach to eliminate infeasibilities regarding time window violations. The performance of the proposed algorithm and the effectiveness of the constraint handling technique have been studied extensively, comparing it to that of state-of-the-art metaheuristics using several sets of benchmark problems reported in the literature. The results of the numerical experiments show that the EMA generates feasible and near-optimal results within shorter computational times compared to the test algorithms.

## 1. Introduction

The traveling salesman problem with time windows (TSPTW) is an important variant of the well-known traveling salesman problem (TSP) in which each customer has a service time (i.e., the time that should be spent during the visit to the customer), which should start within a given time window. In the TSPTW, the time window of each visit is bounded by an earliest arrival time and a latest arrival time. The TSPTW can be defined as the problem of finding a minimum cost tour that starts and ends at the same depot, where each node should be visited exactly once within its time window. A nonnegative cost is associated with each arc, and the total cost can be taken as the route completion time, total travel time, or total traveled distance [1]. In this study, the total traveled distance will be considered the cost value. The TSPTW can be considered a special case of the capacitated vehicle routing problem with time windows (VRPTW) to which the relaxation of capacity constraints is applied.

The TSPTW has various practical real-world applications such as package delivery, school bus routing, scheduling, automated guided machines, and routing problems in the context of lean manufacturing. Savelsbergh [2] has shown that the TSPTW is NP-hard, and even finding a feasible route is a NP-complete problem. Nevertheless, early study focused on exact optimization techniques to solve the TSPTW. Baker [1] proposed an exact algorithm using a branch and bound approach in which lower bounds are determined by dual relaxations of the model. Dumas et al. [3] developed a dynamic programming approach, and Langevin et al. [4] described a two-commodity flow model including two complementary flows. More recently, a branch and cut algorithm [5], linear time dynamic programming [6], and constraint programming [7, 8] were proposed to solve the TSPTW.

Because it is difficult to solve the TSPTW within acceptable computation times using exact methods, Heuristic and metaHeuristic techniques have been analyzed extensively in the literature. Carlton and Wesley Barnes [9] proposed a static-penalty-based tabu search to solve the TSPTW. Gendreau et al. [10] presented an insertion Heuristic, and Calvo [11] used a construction Heuristic based on both greedy insertion and local search. Furthermore, Da Silva and Urrutia [12] proposed a general variable neighborhood search (VNS) Heuristic that consists of a constructive stage for finding a feasible solution and an optimization stage during which feasible solutions are improved using a general VNS Heuristic. Ohlmann and Thomas [13] proposed a compressed annealing approach, which is a variant of simulated annealing and utilizes a variable penalty method, and López-Ibáñez and Blum [14] proposed a Beam-ACO algorithm, which hybridizes Beam search and ant colony optimization to solve the TSPTW via extensive computational analysis.

In this study, an electromagnetism-like algorithm (EMA) using a new variable bounding strategy is proposed to solve the TSPTW efficiently. The EMA approach basically emulates electromagnetism theory in physics, in which charged particles exert attractive or repulsive forces on each other [15]. The basic idea behind the algorithm is to force particles to search for the optimum in a multidimensional space by applying a collective force on them. In recent work, an EMA was used to train neural networks [16] to solve fuzzy relations [17]. Debels et al. [18] solved resource constrained scheduling problems by combining an EMA with scatter search. Tsou and Kao [19] used an EMA to control and optimize multiobjective inventory models. Maenhout and Vanhoucke [20] used an EMA to solve nurse scheduling problems. Jhang and Lee [21] used an EMA for array pattern optimization in the field of electrical engineering. Chang et al. [22] combined a GA with an EMA to solve single machine earliness/tardiness scheduling problems and showed that the hybrid EMA performs better than the plain EMA. Moreover, the researchers used a random key procedure to decode particles into feasible schedules. Wu et al. [23] applied an EMA using a random key procedure to solve the traveling salesman problem. Yurtkuran and Emel [24] solved capacitated vehicle routing problems by using an EMA that hybridizes a local search procedure. Naji-Azimi et al. [25] combined preprocessing procedure, mutation, and local search with an EMA to solve the well-known unicost set covering problem. In [26] EMA framework was introduced for solving the response time variability problem. Guan et al. [27] used an EMA to solve flow path design problems for automated guided vehicles. Jamili et al. [28] proposed a simulated annealing and electromagnetism-like mechanism hybrid framework to solve the periodic job shop scheduling problem. In [29] EMA was used to detect circles on figures. Su and Lin [30] introduced an EMA mechanism for feature selection. Lee and Chang [31] used an improved EMA to optimize fractional-order PID controllers. Furthermore, EMAs have also been applied to address multimode project scheduling under uncertainty [32] and nonlinear system control [33].

In this study, new constraint handling techniques are introduced to cope with time-window constraints in the TSPTW. To the best of our knowledge, our proposed constraint handling technique is the first in the literature in which the feasibility is maintained without using any extra feasibility operators. The main goal of using VBS is to narrow the unbounded search space to a bounded search space to reach feasible solutions effortlessly. Moreover, to analyze the effectiveness of the proposed algorithm, first, the constraint handling performance of the proposed EMA framework is analyzed, comparing it to that of the traditional EMA. Then, the modified framework is compared to state-of-the-art algorithms using benchmark problems.

The rest of this paper is organized as follows.  Section 2 provides a brief introduction and a mathematical formulation of the TSPTW.  Section 3 discusses the traditional EMA, and the proposed EMA for the TSPTW is presented in Section 4. The computational results are summarized in Section 5, and, finally, Section 6 concludes the paper.

## 2. Traveling Salesman Problem with Time Windows

The traveling salesman problem with time windows can be briefly defined as follows. Let *G* = (*N*, *A*) be an undirected complete graph, where *N* = {0,1,…*n*} is the node (customer) set and *A* = {(*a*_*ij*_), *i* = 1,…, *n*, *j* = 1,…, *n*, *i* ≠ *j*} is the arc set. Node 0 denotes the depot, and *N* denotes the number of customers. There is a cost *c*_*ij*_ associated with every arc *a*_*ij*_ ∈ *A*. The cost *c*_*ij*_ generally represents the distance or time between nodes *i* and *j*, plus a service time *s*_*i*_ at customer *i*. In addition, each node *i* ∈ *N* has a time window [*e*_*i*_, *l*_*i*_], where *e*_*i*_ denotes the earliest time and *l*_*i*_ the latest time in which the service can begin. In most of the formulations, waiting times are permitted; that is, arrival to node *i* before *e*_*i*_ is feasible, but a waiting time till *e*_*i*_ is applied. On the other hand, arrival to node *i* after *l*_*i*_ is not permitted. The TSPTW can be mathematically formulated as follows:

decision variables:

*r*_*ij*_: (1) $$\begin{array}{c} r_{ij}=\{\begin{array}{cc} 1, & \text{if}\;\text{arc}a_{ij}\in A\;\text{is}\;\text{used},\\ 0, & \text{otherwise}. \end{array} \end{array}$$
 
*y*_*i*_: position of node *i*, where *i* ∈ *N* within the tour;
 
*t*_*i*_: arrival time at node *i*, *i* ∈ *N*;
 
*w*_*i*_: waiting time at node *i*, *i* ∈ *N*;

parameters:

*N*: set of nodes;
*c*_*ij*_: travel time (distance) from node *i* to *j*, where *i*, *j* ∈ *N*;
*s*_*i*_: service time at node *i*, *i* ∈ *N*;
*e*_*i*_: earliest arrival time at node *i*, *i* ∈ *N*;
*l*_*i*_: latest arrival time at node *i*, *i* ∈ *N*;
  (2)   minimize ⁡∑i∈N∑j∈N(cij+si)rij
  (3)   subject to∑i∈Nrij=1, ∀j∈N,i≠j.
  (4)  ∑j∈Nrji=1, ∀i∈N,j≠i.
  (5) yi−yj+Nrij≤N−1, ∀i,j∈N,  j≠1,  j≠i.
  (6) tj−ti−cij−si−wi≥−M(1−rij),∀i,j∈N,  i≠j.
  (7)  ei≤ti+wi≤li, ∀i∈N.
  (8)  1≤yi≤N, ∀i∈N.
  (9)  ti,wi∈ℝ+ ∀i∈N.

Formula (2) denotes the objective function of the problem. The objective is to minimize the total time required to travel from each node *i* to node *j* and the service time at each node *i*. Constraints (3) and (4) ensure that every node is visited only once. Constraint (5) aims to maintain the sequence of the route. Constraints (6) and (7) specify the service time windows, where *M* denotes a large real number. Constraint (8) bounds the sequence *y*_*i*_ for each node *i*. Depending on the tight bounds in time-window constraints (7), TSPTW results in a tighter search space of feasible solutions. If an efficient neighborhood search strategy which is part of a metaHeuristic solver takes advantage of this feasible solution space, it is expected that the performance of such a metaHeuristic algorithm can be improved. Therefore, in the following sections, EMA will be used to host our proposed strategy for handling constraint (7).

## 3. Electromagnetism-Like Algorithm

The EMA is a population-based Heuristic method introduced by Birbil and Fang [15] for solving bound constraint optimization problems. The algorithm was inspired by the theory of electromagnetism in physics, in which there are attractive and repulsive forces between charged particles. The EMA can be used easily and effectively to solve optimization problems with bounded variables of the following form:

(10) minimize f(x)subject to x∈[L,U],

where [**L**, **U**] = {**x** ∈ ℝ^*n*^ | *L*_*k*_ ≤ *x*_*k*_ ≤ *U*_*k*_, *k* = 1,…, *m*}.

In the problem formulation, **x** is a vector that represents a solution point position in *m*-dimensional space in a population of *n* position vectors. *x*_*k*_ denotes the variable of the *k*th dimension (i.e., axis). Each variable *x*_*k*_ has an upper and lower bound, *U*_*k*_ and *L*_*k*_, respectively, and *f*(**x**) indicates the objective function value (OFV) of the candidate solution **x**.

In the EMA, a candidate solution is associated with a charged particle in a multidimensional space using a real-coded position vector **x**. Index *k* in each particle *i*'s position vector **x**_*i*_ identifies a dimensional element *x*_*ik*_, where *k* = 1,…, *m* and *i* = 1,…, *n*. In other words, *n* and *m* are the population size and the total number of variables, respectively. The OFV of the *i*th candidate solution is calculated by using its position vector. The charge of particle *i*, *q*_*i*_, depends on the quality of the OFV. The better the OFV of the particle is, the greater amount of charge the particle has. Each particle exerts a repulsive or attractive force on other population members according to the charges they carry. The resultant force **F**_*i*_ is determined by calculating the vector sum of the forces exerted on a particle *i*. Then, **x**_*i*_ is updated by **F**_*i*_ at each iteration. The key idea of the EMA is that, for a minimization problem, a candidate particle *i* will attract particle *j* if particle *i* has a better OFV than particle *j* (i.e., *f*(**x**_*i*_) < *f*(**x**_*j*_)), whereas if*f*(**x**_*j*_) < *f*(**x**_*i*_), particle *i* will repel particle*j*.

The traditional EMA has four phases: (1) initialization, (2) calculation of particle charges and force vectors, (3) movement according to the resultant force, and (4) local search to exploit the local minima [15]. The general scheme of the algorithm is presented in Algorithm 1 (for more details, readers are referred to [15]). In Algorithm 1, *PopSize* represents the population size, and *MaxIter* and *LsIter*  are the maximum iteration number for the algorithm and the local search procedure, respectively. An *Initialize*() procedure is used to generate *PopSize* number of points randomly from the search space. A *LocalSearch*() procedure is applied to the particles to improve the solution quality and to force the algorithm to search for unvisited regions. Then, *CalculateCharges*(), *CalculateForces*(), and *Movement*() procedures are applied to the particles within the population at each iteration.

In this study, the charge and force calculations and the subsequent particle movement procedures are implemented using the modified EMA proposed by [18]. Here, the charge and force calculations are not absolute-value-based; instead, relative charge and force calculations are used for each particle pair in the population. The details of the proposed algorithm will be described in the next section.

## 4. EMA for TSPTW

This section provides the details of the proposed EMA.

### 4.1. Charge and Force Calculations and Movement of Particles

In our proposed EMA, the charge of particle *i* is defined as *q*_*ij*_, which is relative to that of particle *j*, whereas it has been defined as *q*_*i*_ in previous works [20, 21, 25, 26, 29, 32, 33]. The value of *q*_*ij*_ can be obtained by calculating the relative difference between the OFVs of particles *i* and *j* [18]:

(11) $$\begin{array}{c} q_{ij}=\frac{f\left(x_{i}\right)-f\left(x_{j}\right)}{f\left(x_{\text{worst}}\right)-f\left(x_{\text{best}}\right)}\;\forall i,j\in N,\;\;i\neq j, \end{array}$$

where *f*(**x**_worst_) and *f*(**x**_best_) are the worst and best OFVs in the population, respectively. For a minimization problem, if *f*(**x**_*i*_) < *f*(**x**_*j*_), then *q*_*ij*_ will be negative, and the reverse is true if *f*(**x**_*i*_) > *f*(**x**_*j*_). If *f*(**x**_*i*_) = *f*(**x**_*j*_), then *q*_*ij*_ will be zero.

After calculating *q*_*ij*_, the force vector *F*_*ij*_ exerted on particle *i* by particle *j* is calculated as follows:

(12) $$\begin{array}{c} F_{ij}=\left(x_{j}-x_{i}\right)q_{ij},\;\forall i,j\in N,\;\;i\neq j. \end{array}$$

For a minimization problem, if particle *i* is a better solution than particle *j*, that is, *f*(**x**_*i*_) < *f*(**x**_*j*_), then particle *j* will repel particle*i* because *q*_*ij*_ will be negative.

In the modified EMA, first, *q*_*ij*_ and *F*_*ij*_ are calculated for every combination of particle pairs; then, the resultant cumulative force on particle *i* is determined by **F**_*i*_ = ∑_*j*=1,*j*≠*i*_^*n*^ *F*_*ij*_. Once the cumulative force **F**_*i*_ exerted on particle *i* is determined, the particle *i* will move in the direction of **F**_*i*_ to yield improved solutions. A uniformly distributed random step 0 < *λ* < 1 is used to force the algorithm to explore unvisited regions. Similarly to the cooling effect in the simulated annealing algorithm, the current iteration number, *Iter*, is used to decrease the step length as the algorithm proceeds. Additionally, a preset parameter *r*, 0 ≤ *r* ≤ 1, is used to control the cooling effect. The motion of particle *i* along the **F**_*i*_ direction is calculated as follows:

(13) $$\begin{array}{c} x_{i}=x_{i}+\frac{\lambda }{{\left(Iter\right)}^{r}}F_{i},\;\forall i\in N. \end{array}$$

It is important to note that the proposed EMA uses an elitist strategy. In other words, the best solution's position vector in the population, **x**_best_, is preserved.

### 4.2. Solution Representation

As mentioned above, the EMA was originally designed to cope with continuous optimization problems. In order to solve combinatorial optimization problems such as vehicle routing problems with EMA, real-coded position vectors (candidate solutions) have to be decoded into permutations of customers. To the best of our knowledge, most researchers have introduced a random key (RK) procedure into the EMA to facilitate solving combinatorial optimization problems [18, 22, 25, 26].

**Figure 1:**
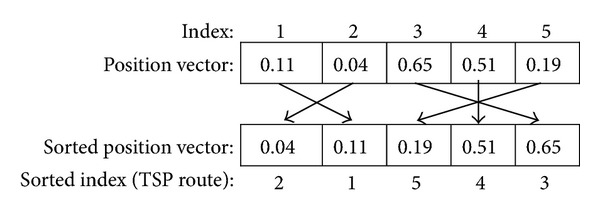
A sample random key procedure.

The RK representation was proposed by [34] to maintain feasible solutions after crossover operations in genetic algorithms. In [34] a random number encoding structure was proposed for the chromosomal representation of solutions. The main advantage of using the RK procedure is that each candidate solution can be represented by real-coded values such that several metaHeuristic operators can be implemented without concern over feasibility issues. Because all position vectors are real-coded, integrating the random key procedure into the EMA is a very straightforward and easy process, thus making the EMA an efficient search algorithm for combinatorial optimization problems. A sample random key procedure is shown in Figure 1. In the random key procedure, when the real-coded coordinate values of the position vector are sorted in a nondecreasing order, the new permutation of the indexes of this position vector represents a route for the TSPTW as a sorted index. In Figure 1, because the smallest coordinate value of the position vector is 0.04 for index = 2, customer 2 will be visited first. The other customers are visited following the sorted index in a similar manner, and the resulting route will be 2→1→5→4→3.

### 4.3. Handling Time Window Constraints

In the proposed EMA, two approaches are combined to prevent infeasible routes: (i) new variable bounding strategy (VBS) and (ii) penalty approach. VBS is used to eliminate infeasible candidate solutions when the problem consists of *nonoverlapping time windows* for nodes, whereas a penalty strategy is used to cope with infeasibilities resulting from *overlapping time windows*.

#### 4.3.1. VBS with Nonoverlapping Time Windows

**Figure 2:**
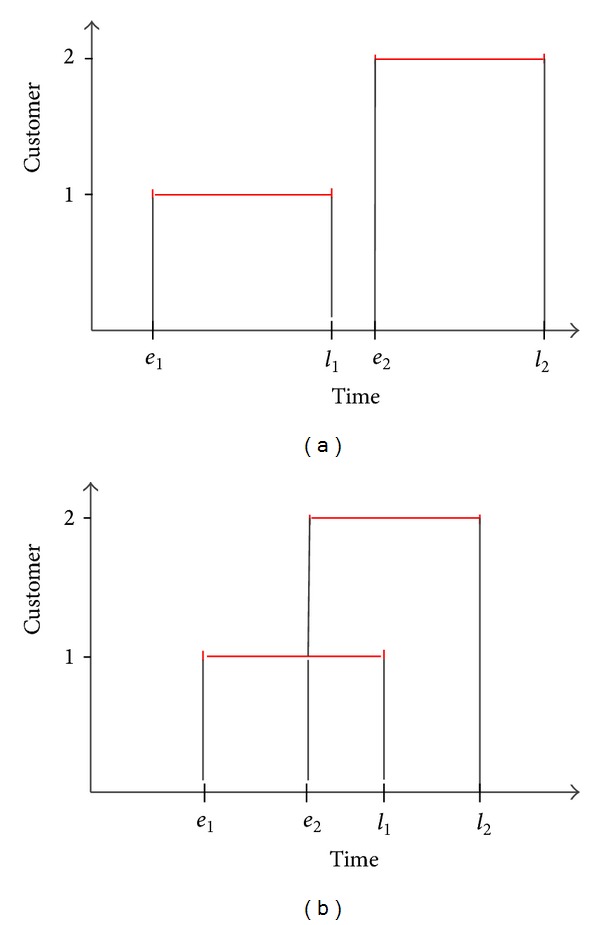
Time windows for a customer pair: (a) nonoverlapping time windows and (b) overlapping time windows.

Nonoverlapping time window infeasibilities can be described as follows. Consider two customers *i* and *j* with time windows [*e*_*i*_, *l*_*i*_] and [*e*_*j*_, *l*_*j*_], respectively. These time windows are said to be nonoverlapping if and only if either *l*_*i*_ ≤ *e*_*j*_ or *l*_*j*_ ≤ *e*_*i*_, where *e*_*i*_ ≤ *l*_*i*_ by definition (Figure 2(a)). Because a waiting time up to *e*_*i*_ is applied for early visits, the earliest time (*t*_*i*_^earliest^) in which a customer can be left is the early time of that customer (*t*_*i*_^earliest^ ≥ *e*_*i*_). Therefore, any customer sequence that ensures the following constraint will always be infeasible for a nonoverlapping customer pair:

(14) $$\begin{array}{c} e_{i}+s_{i}+d_{ij}\geq l_{j},\;\left(i,j\right)\in s,\;\;d_{i,j}>0, \end{array}$$

where *s* represents the set of customer pairs that customer *i* precedes *j*. In other words, if *l*_*i*_ < *e*_*j*_, then customer *i* should be visited before customer *j*; otherwise, if *l*_*j*_ < *e*_*i*_, then customer *j* should be visited before customer *i* and any tour that contains a sequence in which *j* is visited before customer *i* is infeasible.

VBS relies on the ability of the EMA to operate with bounded variables. In VBS, the time windows of customers are normalized between predetermined lower and upper global bounds [*L*, *U*], and the variables are then bounded within their corresponding normalized time windows [*e*_*k*_^nor^, *l*_*k*_^nor^], where *e*_*k*_^nor^ = *e*_*k*_(*L*/min⁡*E*), *l*_*k*_^nor^ = *l*_*k*_(*U*/max⁡*L*), min⁡*E* = min⁡_*k*_⁡{*e*_*k*_}⁡, and max⁡*L* = max⁡_*k*_⁡{*l*_*k*_} and *k* represents the customer number. Combining the VBS and the nondecreasing sorting step in RK, any solution point that has infeasible customer pairs with nonoverlapping time windows is thus eliminated from the candidate solution population. In other words, EMA is forced to search in feasible regions using VBS.

#### 4.3.2. Penalty Strategy with Overlapping Time Windows

However, this variable bounding strategy has drawbacks in the case of highly overlapping time windows. The effect of VBS will decrease in going from nonoverlapping to overlapping windows and will be ineffective if the time windows of a customer pair (*i*, *j*) are fully overlapping (*e*_*i*_ = *e*_*j*_ and  *l*_*i*_ = *l*_*j*_) (see Figure 2(b) for an overlapping time window example). To overcome the infeasibility problems for highly overlapping time windows, a penalty strategy is introduced. A penalty cost that is calculated from a linear penalty function is added to the OFV if the solution violates the time window of any customer. This penalty is assumed to be a linear function of the amount of time that is violated. The penalty cost is calculated as follows:

(15) $$\begin{array}{c} P_{i}=\{\begin{array}{cc} 0 & t_{i}<e_{i}\\ 0 & e_{i}{\leq t}_{i}\leq l_{i}\\ \beta \left(t_{i}-l_{i}\right) & t_{i}>l_{i}, \end{array} \end{array}$$

where *P*_*i*_ denotes the penalty cost at customer *i*, *t*_*i*_ represents the vehicle arrival time to customer *i*, and *β* is the penalty coefficient, which will be determined experimentally. By using the penalty approach, infeasible solutions will have high OFVs and will exert repulsive forces on better solutions.

**Figure 3:**
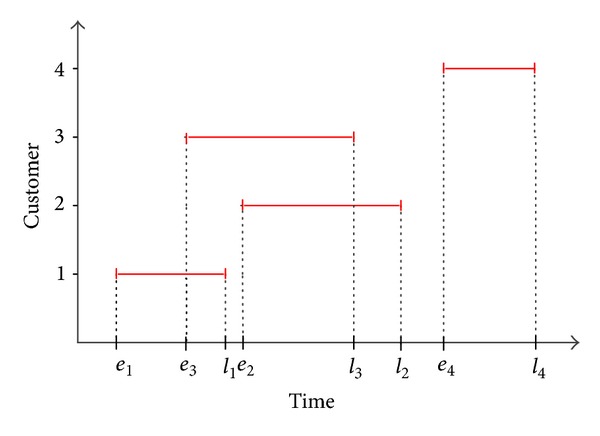
Time windows for the sample problem.

**Figure 4:**
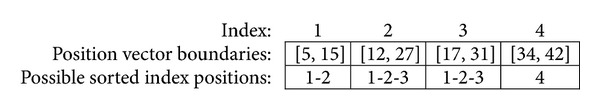
Possible routes after combining VBS and RK.

The effect of VBS and RK in eliminating time window infeasibilities is illustrated by considering a sample problem. Consider a TSP with 4 customers having time windows, as indicated in Table 1. For ease of analysis, we ignore the normalization step and time windows are directly used as the bounds. As shown in Figure 3, customers (1,2), (1,4), (2,4), and (3,4) have nonoverlapping time windows. By definition, customer 4 should be visited last because *e*_4_ ≥ *l*_*j*_, *j* = 1,2, 3. Because we bound index 4 of the position vector with customer 4's normalized time window [*e*_*k*_^nor^, *l*_*k*_^nor^], index 4 will always be larger than the other variables during the search process. As a result, when the real-coded coordinate values of the position vector are sorted in a nondecreasing order in RK, it will be ensured that customer 4 will be the last. Similarly, by combining VBS and RK, customer 1 will always precede customer 2 because *e*_2_ ≥ *l*_1_. To summarize, by combining VBS and RK as a solution representation strategy, infeasibilities associated with nonoverlapping time windows are eliminated, and those infeasible regions are abandoned.  Figure 4 shows the variable boundaries and possible positions of customers after using the VBS and RK. Furthermore, a penalty approach will help to discern feasible solutions from the possible feasible solution set because infeasible solutions will have higher OFVs.

### 4.4. Initialization and Boundary Control

The EMA framework begins with the initialization mechanism. The *Initialize*() procedure generates *PopSize*() solutions as a starting population using the normalized time windows. The procedure is shown in Algorithm 2, where *n* and *m* denote the population size and length of the position vector (i.e., number of variables), respectively, and *Uniform*() draws samples from a uniform distribution. Each position vector **x**_*i*_ is initialized randomly within the time window of each corresponding variable, **x**_*ik*_.

At the end of each iteration, boundary control is applied to the position vector of the particles to determine whether any boundary violations occur. The proposed algorithm adopts an absorbing bound-handling scheme, where, in the case of boundary violation, the corresponding variable is relocated to the bound.  Algorithm 3 is the boundary control mechanism; as indicated, if the particle flies outside the boundary, the corresponding variable is set to its normalized early or late arrival time.

### 4.5. General Scheme of EMA

The general scheme of the proposed EMA for solving the TSPTW is summarized in Algorithm 4. Each step is executed according to the explanations provided above.

## 5. Computational Results

The proposed algorithm, described in the previous sections, was implemented in Visual Basic. Net on a PC with an Intel Core 2 Duo CPU running at 2.0 GHz with 2 GB RAM for computational experiments. Two types of experiments were carried out to assess the effectiveness of the proposed EMA. First, to evaluate the performance of the VBS, the EMA with VBS and that without VBS are compared with respect to selected benchmark instances. Second, the proposed EMA is compared to state-of-the-art metaHeuristics using an extensive set of benchmark instances reported in the literature.

### 5.1. The Effectiveness of Variable Bounding Strategy for the TSPTW

To understand the role of VBS in finding feasible solutions, the proposed EMA with VBS and a penalty strategy (EMA-VP) was compared with the EMA with only a penalty strategy (EMA-P). Because the level of time window overlapping is the key criterion in analyzing the effect of VBS, six different problem instances selected from the benchmark set provided by Potvin and Bengio [35] are categorized as exhibiting a *low*, *average,* or *high* level of time window overlapping. An explicit indicator value of the overlap level (VOL) is calculated by adding two percentages calculated from problem instances, that is, (i) the percentage of time windows of two or more customers that intersect over the total time line (min⁡*E*  and  max⁡*L*) and (ii) the ratio of customers with an overlapping time window of at least one unit length. In other words, the length of the overlapped time and number of customers having overlapping time windows are calculated as basic indicators. Therefore, a VOL of 200 corresponds to the full overlap of time windows, whereas 0 denotes a nonoverlapping problem.

**Figure 5:**
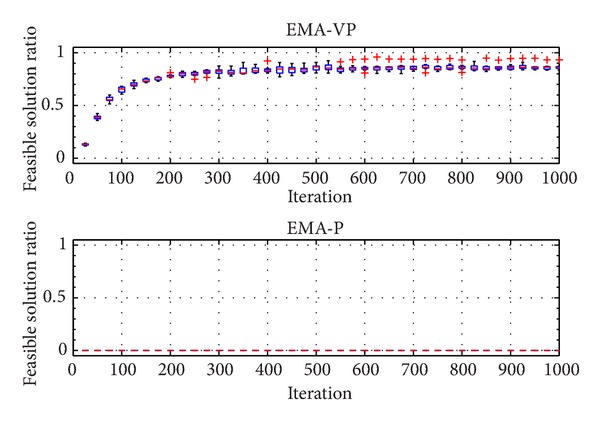
Ratio of feasible solutions for EMA-VP and EMA-P for RC 201.3.

**Figure 6:**
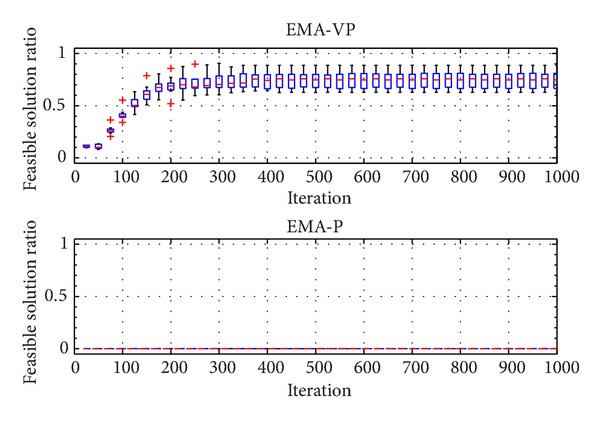
Ratio of feasible solutions for EMA-VP and EMA-P for RC 202.1.

**Figure 7:**
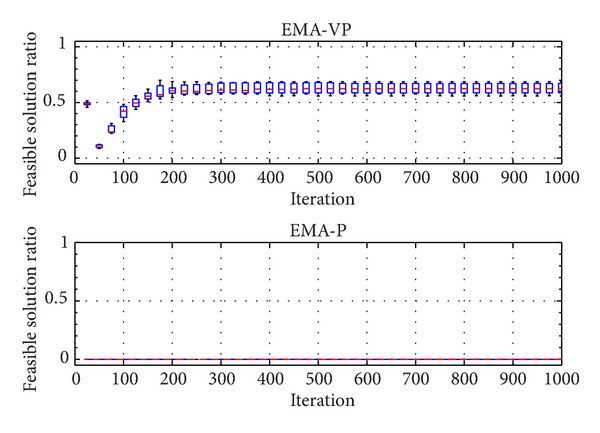
Ratio of feasible solutions for EMA-VP and EMA-P for RC 203.2.

**Figure 8:**
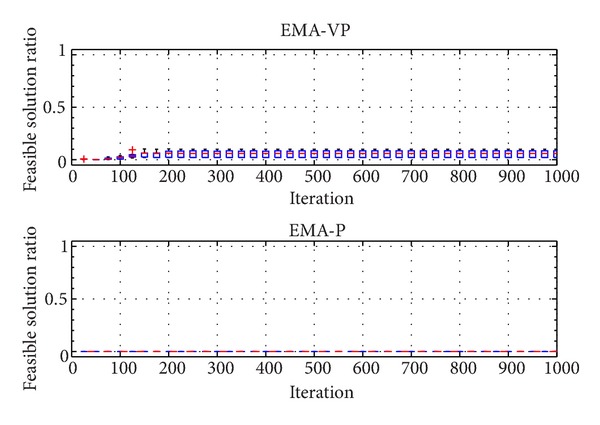
Ratio of feasible solutions for EMA-VP and EMA-P for RC 204.2.

**Figure 9:**
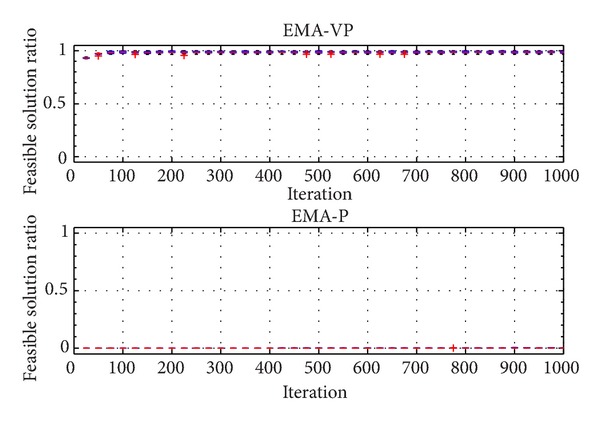
Ratio of feasible solutions for EMA-VP and EMA-P for RC 205.1.

**Figure 10:**
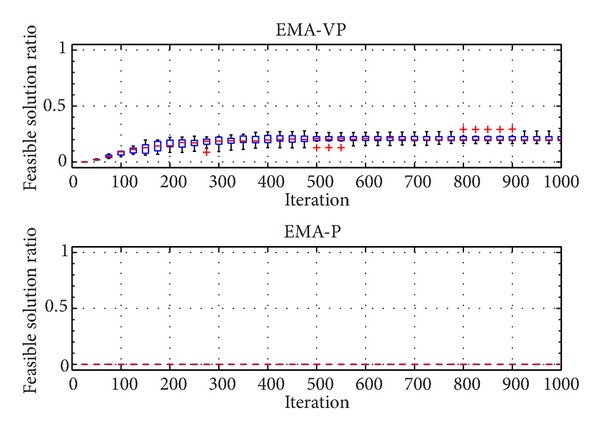
Ratio of feasible solutions for EMA-VP and EMA-P for RC 208.1.

The categorized VOLs are presented in Table 2. Furthermore, Table 3 summarizes the selected problem parameters and the corresponding VOLs. Problems with a similar number of customers from different classes (RC 201.3, RC 202.1, RC 203.2, and RC 204.2), a small problem with an average VOL (RC 205.1), and a relatively more complex problem (RC 208.1) are selected for the experiments. In Table 3, *n* denotes the total number of customers and the depot. Figures 5, 6,7, 8, 9, and 10 show the ratio of the number of feasible solutions to the total population as the algorithm proceeds from each benchmark problem. For those experiments, the population size is set to 25 and the penalty coefficients *β* and *r* are set to 1.5 and 0.35, respectively. A single run represents 1000 iterations, and the ratio of feasible solutions is recorded at the end of 25 iterations. Box plots show 10 independent data, each of which represents an average of 25 consecutive runs.

The experiments show that the EMA-VP clearly outperforms EMA-P (Figures 5–10). The following conclusions can be drawn from the figures. (1) In all six experiments, the EMA-VP demonstrates better performance than the EMA-P as expected. (2) In all six experiments, EMA-VP finds feasible solutions, whereas the EMA-P finds a limited number of feasible solutions only for the smallest problem (RC 205.1). (3) Except for the benchmarks with high VOL (RC 204.2 and RC 208.1), the VBS generates a feasible initial population with a minimum ratio of 0.1, whereas the EMA-P always starts searching with an infeasible initial population. (4) The EMA-VP quickly converges to a significant ratio of feasible solutions in the first 150–250 iterations and finds a maximum of 100% (RC 205.1) and a minimum of 3% (RC 204.2) feasible solutions in the final populations. (5) The negligible reduction (clearly observed for benchmark problem RC 203.2, Figure 7) in the ratio of feasible solutions in the first 50 iterations indicates that particles are generally stagnant at the bounds under high magnitudes of resultant forces. Particles are more likely to leave the search space because relatively high resultant force values are applied in the early phases of the searching process as force magnitudes are reduced iteratively (see (13), Section 4.1).

### 5.2. Comparison Using Benchmark Instances

To verify the effectiveness of the proposed algorithm, we performed an extensive analysis using several benchmark instances. The results of this study are presented in a manner similar to that of state-of-the-art studies by López-Ibáñez and Blum [14] and Ohlmann and Thomas [13] for an accurate and objective comparison. The three benchmark sets used to test the proposed EMA-VP mechanism are as follows.

1. *Benchmark Set 1*. The benchmark set was introduced by Potvin and Bengio [35], which was originally developed for VRPTW problems by Solomon [36]. This set includes 29 problems and is the most widely used benchmark set for the TSPTW. Originally, the set included 30 problems; however, there was a conflict among the researchers about the node number and best solution value for problem RC 204.1 [13, 14]. (Thus, it is not considered here.) (See Table 4.)
2. *Benchmark Set 2*. The set of 70 problems in seven instance classes was introduced by Langevin et al. [4]. These instances range from 20 to 60 customers (Table 5).
3. *Benchmark Set 3*. The set of benchmark instances was introduced by Dumas et al. [3] This set consists of 135 instances, and the customer numbers range from 20 to 200 (Table 6).

Because parameter calibration is the key task in metaHeuristic applications, we performed a set of pilot studies to determine a good set of parameters for the EMA-VP mechanism. After these preliminary computational studies, the parameters were set as follows: *MaxIter* = 500, *β* = 1.5, *r* = 0.35, and *PopSize* = 50. The results are presented in terms of relative percentage deviation (RPD), which is calculated as 100 × (EMA  value − the  Best  known  value)/(the  Best  known  value). Because the EMA (particularly the movement procedure) is stochastic in nature, each result is reported as the average of 10 runs. As presented in the studies by [13, 14], both the mean *μ*_RPD_ and standard deviation *σ*_RPD_ of relative percentage deviation results and the mean CPU times (second) *μ*_*T*_ are reported here as well.

The results of the comparison between the EMA-VP and novel metaHeuristics for the benchmark set 1 are summarized in Table 4. The first column of Table 4 represents the problem name; *n* denotes the number of customers in the problem; VOL indicates the value of the time window overlap level and BKV is the best known solution value of the problems presented in the literature. Furthermore, whereas bold-typed values of BKV are the optimal values reported by others, the values indicated by an asterisk are the optimal values determined using CPLEX 12.1 in this study. The EMA-VP is compared to the Beam-ACO [14], compressed annealing (CA) proposed by [13], dynamic programming (DP) [6], and the best values reported in the studies by Gendreau et al. [10] and Calvo [11] as Heuristic. As shown in Table 4, the EMA-VP finds the optimal or the best known values for 19 out of 30 instances without any solution value variability. The Beam-ACO outperforms the EMA-VP in some of the *high* VOL instances; nevertheless, the differences between the mean RPDs (i.e., RC 204.2, RC 204.3, and RC 208.3) are quite small. Moreover, the EMA-VP and CA yield very similar results in all of the instances, and the results that are obtained by the EMA-VP are better than those obtained by DP and Heuristic. Furthermore, the EMA-VP is able to find a feasible solution for all instances.

The results of the benchmark set 2 are presented in Table 5. These results are the averages of 10 instances of 10 runs, as in the other studies performed by [13, 14]. The EMA-VP is compared to compressed annealing (CA) [13], Beam-ACO [13], and the best known values (BKV) [36]. The EMA-VP yields promising results and the optimal values for the first 4 instances (i.e., *n* = 20 and *n* = 40) and achieved the best known values for the large instances (i.e., *n* = 60). Moreover, no variance in the solution quality is reported. As a result, the EMA-VP is compatible with CA and Beam-ACO for the benchmark set 2. It is important to note that all of the instances in the problem set are *low* and *average* VOLs; thus, the EMA-VP shows promising convergence rates.

Table 6 reports the results of the EMA-VP for the benchmark set 3. The results are presented as averages of 10 instances in each class and 10 runs for each instance. Table 6 compares the EMA-VP with the exact method developed by Dumas et al. [3], Beam-ACO [14], and CA [13], and the last column is titled Heuristic, which represents the best value obtained by the algorithms developed by [9–11]. The EMA-VP yields the optimal values in 19 out of 27 instances. The EMA-VP yields results that are better than those of CA and Heuristic and similar to those of Beam-ACO. EMA-VP outperforms all other algorithms on instances (*n* = 60 and TW = 100) and (*n* = 80 and TW = 60). Moreover, the EMA-VP surpasses Beam-ACO in instances (*n* = 150 and TW = 20 and TW = 40) with respect to both solution quality and consistency. On the other hand, Beam-ACO is slightly better than the EMA-VP in instances (*n* = 80 and TW = 80), (*n* = 200 and TW = 20), and (*n* = 150 and TW = 60).

For a better evaluation of metaHeuristic algorithms, not only the solution quality but also the computation times should be investigated. However, it is not very easy to make an objective comparison between metaHeuristics because both the programming languages and the machine configurations are generally not comparable, and, in most studies, the complexities of the algorithms are not reported. Nevertheless, an approximate comparison can be made based on the MFlop (million floating point operations per second) values of the processors on which the algorithms were coded and run [37].

The CA algorithm was coded in C++ and implemented on an Intel Pentium 4 CPU operating at 2.66 GHz [13], Beam-ACO was implemented in C++ on an Intel Xeon X3350 processor with a 2.66 GHz CPU [14], and Heuristic [11] was coded in FORTRAN and executed on an Intel 486 CPU operating at 66 MHz. The MFlop values of the processor speeds based on the benchmark values obtained from the site http://www.netlib.org/benchmark/linpackjava/timings_list.html and the reported and normalized mean CPU times on the benchmark sets for the algorithms are summarized in Table 7. DP [6] is not included in the comparison because the CPU was not reported in the study. As shown in Table 7, the EMA-VP is faster than Beam-ACO and Heuristic for all of the benchmarks. However, the performances of Heuristic and the EMA-VP are very similar on the benchmark set 1. Moreover, CA is faster than the EMA-VP on benchmark set 3, and EMA-VP outperforms CA on the other benchmark sets. Table 7 clearly shows that, in general, the proposed EMA-VP is an effective algorithm and outperforms other novel algorithms in 9 out of 11 test cases in terms of computational time.

## 6. Conclusion

This paper has presented an EMA with a variable bounding strategy (VBS) as a novel constraint handling technique for solving the traveling salesman problem with time windows. The EMA is an easy-to-code, straightforward metaHeuristic algorithm that emulates the attraction-repulsion interactions of charged particles in analogy to Coulomb's law in electromagnetic theory. The proposed algorithm uses two important approaches to handle time-window constraints, the penalty approach and VBS. VBS is one of the main contributions of this study, in which the upper and lower bounds of a variable are set using the corresponding time window for serving a customer. The main goal of using VBS is to narrow the unbounded search space to a bounded search space to reach feasible solutions effortlessly. We clearly show that our approach competes other approaches reported in the literature. An extensive computational analysis using well-known benchmark instances shows that the EMA-VP converges to feasible regions in a search space and finds the best known or near-optimal results. Furthermore, the EMA-VP outperforms other novel metaHeuristics in terms of computational time. Future work may involve combining the VBS technique with other metaHeuristics using real-coded particles as in particle swarm optimization, differential evolution, or artificial bee colony algorithms to solve combinatorial optimization problems that have constraints similar to time windows, such as scheduling with precedence constraints or resource constraint project management.

## Figures and Tables

**Algorithm 1:**
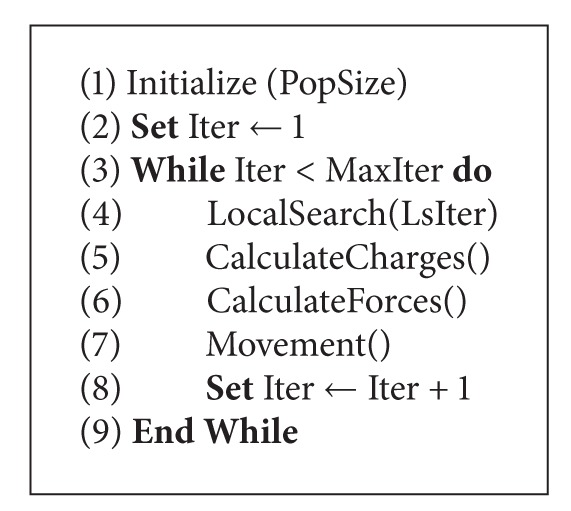
EMA (PopSize, MaxIter, and LsIter).

**Algorithm 2:**
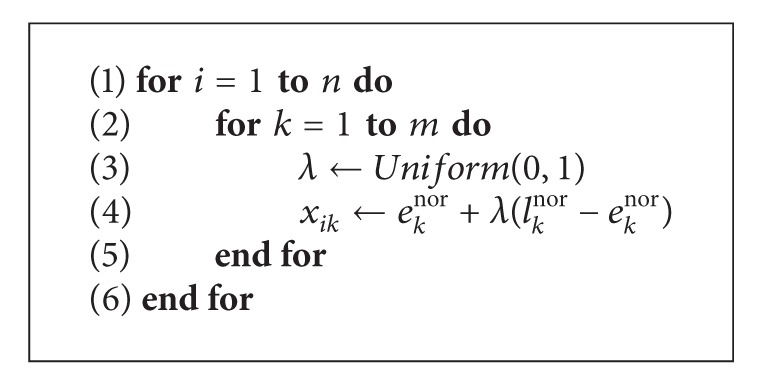
Initialization mechanism.

**Algorithm 3:**
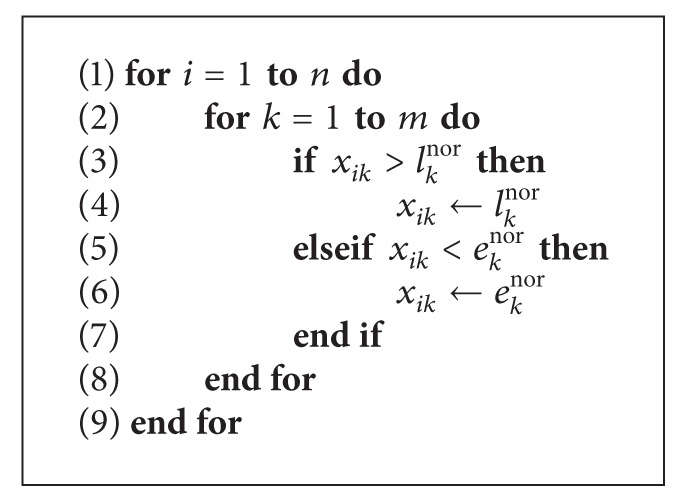
Boundary control mechanism.

**Algorithm 4:**
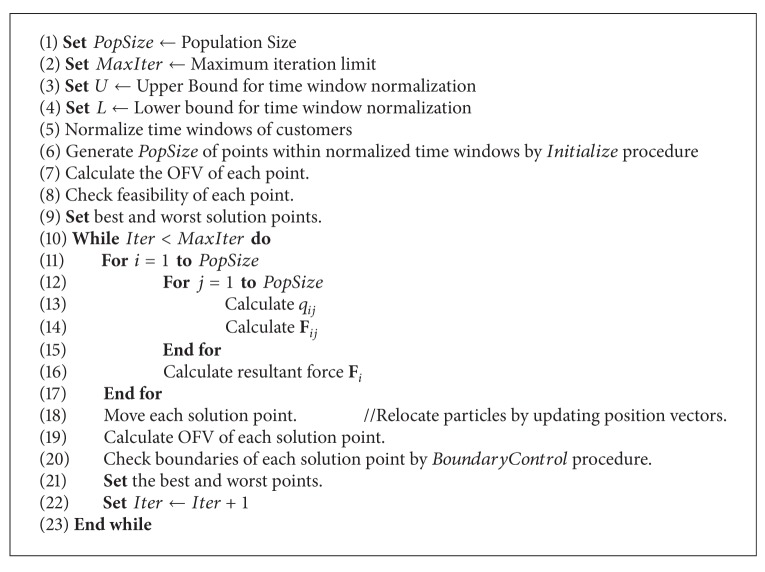
General scheme of proposed EMA.

**Table 1:** Customers' time windows for the sample problem.

Customer	Early time	Late time
1	5	15
2	12	27
3	17	31
4	34	42

**Table 2:** Overlap level classification.

Classes of Overlap Level	VOL
Low	≤90
Average	90 < VOL ≤ 150
High	>150

**Table 3:** Selected problem parameters.

Instance	*n *	VOL	Class of Overlap Level
RC 201.3	32	76.33	Low
RC 202.1	33	107.79	Average
RC 203.2	33	137.72	Average
RC 204.2	33	171.29	High
RC 205.1	14	101.95	Average
RC 208.1	36	161.07	High

**Table 4:** Computational results on benchmark Set 1.

Problem name	*n*	VOL	BKV	EMA-VP			Beam-ACO			CA			DP		Heuristic	
				*μ*_RPD_	*σ*_RPD_	*μ*_*T*_	*μ*_RPD_	*σ*_RPD_	*μ*_*T*_	*μ*_RPD_	*σ*_RPD_	*μ*_*T*_	*μ*_RPD_	*μ*_*T*_	*μ*_RPD_	*μ*_*T*_
RC 201.1	20	86.16	**444.54**	0.00	0.00	1	0.00	0.00	0	0.00	0.00	5	0.00	2	0.00	0
RC 201.2	26	77.92	**711.54**	0.00	0.00	1	0.00	0.00	2	0.00	0.00	6	0.00	3	0.00	0
RC 201.3	32	76.33	**790.61**	0.00	0.00	2	0.00	0.00	0	0.00	0.00	9	0.00	4	0.00	3
RC 201.4	26	79.20	**793.64**	0.00	0.00	1	0.00	0.00	0	0.00	0.00	6	0.00	3	0.00	0

RC 202.1	33	107.79	771.78	0.21	0.06	3	0.00	0.00	0	0.05	0.02	11	0.07	223	0.05	8
RC 202.2	14	153.95	**304.14**	0.00	0.00	0	0.00	0.00	0	0.00	0.00	5	0.00	2	0.00	0
RC 202.3	29	84.49	**837.72**	0.00	0.00	1	0.00	0.00	1	0.00	0.00	7	0.00	45	0.22	0
RC 202.4	28	115.19	793.03	0.00	0.00	1	0.00	0.00	0	0.00	0.00	9	0.78	212	0.00	2

RC 203.1	19	133.88	**453.48**	0.00	0.00	0	0.00	0.00	0	0.00	0.00	7	0.00	15	0.00	0
RC 203.2	33	137.72	784.16	0.00	0.00	4	0.00	0.00	0	0.00	0.00	11	3.14	404	0.00	4
RC 203.3	37	142.48	817.53	0.03	0.01	3	0.00	0.00	2	0.03	0.11	12	infeasible		0.23	14
RC 203.4	15	171.69	**314.29**	0.00	0.00	0	0.00	0.00	0	0.00	0.00	5	0.00	3	0.00	0

RC 204.2	33	171.29	662.16	0.31	0.14	3	0.00	0.00	8	0.71	1.29	10	0.00	77	0.57	8
RC 204.3	24	183.24	455.03*	0.74	0.10	3	0.00	0.00	0	0.96	0.50	9	2.46	639	0.00	4

RC 205.1	14	101.95	**343.21**	0.00	0.00	0	0.00	0.00	0	0.00	0.00	4	0.00	2	0.00	0
RC 205.2	27	93.19	**755.93**	0.00	0.00	2	0.00	0.00	0	0.00	0.00	7	0.00	5	0.00	0
RC 205.3	35	114.92	825.06	0.00	0.00	4	0.00	0.00	1	0.00	0.00	10	0.00	42	0.00	21
RC 205.4	28	89.68	**760.47**	0.00	0.00	3	0.00	0.00	5	0.00	0.00	7	0.00	5	0.26	6

RC 206.1	4	156.35	**117.85**	0.00	0.00	0	0.00	0.00	0	0.00	0.00	3	0.00	0	0.00	0
RC 206.2	37	109.01	828.06	0.00	0.00	5	0.00	0.00	0	0.01	0.04	11	0.00	33	1.70	33
RC 206.3	25	122.48	574.42*	0.00	0.00	1	0.00	0.00	1	0.00	0.00	9	0.00	38	0.00	0
RC 206.4	38	108.79	831.67	0.04	0.01	5	0.00	0.00	3	0.10	0.24	11	0.00	46	0.71	8

RC 207.1	34	127.74	732.68	0.09	0.03	5	0.00	0.00	0	0.00	0.00	11	0.43	70	0.07	4
RC 207.2	31	136.27	701.25	0.00	0.00	5	0.00	0.00	7	0.00	0.00	10	0.00	61	2.40	16
RC 207.3	33	139.30	616.51	0.44	0.11	5	0.00	0.00	1	0.00	0.00	11	2.28	1128	0.29	17
RC 207.4	6	165.89	**612.85**	0.00	0.00	0	0.00	0.00	0	0.00	0.00	3	0.00	0	0.00	0

RC 208.1	38	161.07	605.54	0.33	0.10	5	0.30	0.29	19	0.58	0.36	12	0.55	1141	0.00	10
RC 208.2	29	165.56	**601.89**	0.18	0.02	4	0.00	0.00	1	0.17	0.54	10	0.00	59	0.67	2
RC 208.3	36	164.94	598.24	1.01	0.04	5	0.00	0.00	12	0.95	0.84	11	3.32	122	2.31	8

**Table 5:** Computational results on benchmark set 2.

Data set		VOL	BKV	*T* cpu	EMA-VP			Beam-ACO			CA		
*n*	TW width				*μ*_RPD_	*σ*_RPD_	*μ*_*T*_	*μ*_RPD_	*σ*_RPD_	*μ*_*T*_	*μ*_RPD_	*σ*_RPD_	*μ*_*T*_
20	30	48.51	**724.7**	0	0.00	0.00	1	0.00	0.00	0	0.00	0.00	5
	40	53.60	**721.5**	1	0.00	0.00	1	0.00	0.00	0	0.00	0.00	5

40	20	40.81	**982.7**	2	0.00	0.00	5	0.00	0.00	0	0.00	0.00	7
	40	51.22	**951.8**	7	0.00	0.00	5	0.00	0.00	0	0.00	0.00	7

60	20	40.33	1215.7	—	0.00	0.00	7	0.00	0.00	0	0.00	0.00	9
	30	45.48	1183.2	—	0.00	0.00	8	0.00	0.00	0	0.00	0.00	12
	40	49.12	1160.7	—	0.00	0.00	8	0.00	0.00	3	0.00	0.01	14

**Table 6:** Computational results on benchmark set 3.

Data set			Exact		EMA-VP			Beam-ACO			CA			Heuristic	
*n*	TW. width	VOL	Optimal value	*T*	*μ*_RPD_	*σ*_RPD_	*μ*_*T*_	*μ*_RPD_	*σ*_RPD_	*μ*_*T*_	*μ*_RPD_	*σ*_RPD_	*μ*_*T*_	*μ*_RPD_	*μ*_*T*_
20	20	106.17	**361.2**	0	0.00	0.00	1	0.00	0.00	0	0.00	0.00	5	0.00	0
	40	87.56	**316.0**	0	0.00	0.00	1	0.00	0.00	0	0.00	0.00	5	0.00	0
	60	86.78	**309.8**	0	0.00	0.00	1	0.00	0.00	0	0.00	0.00	5	0.00	0
	80	76.96	**311.0**	0	0.00	0.00	1	0.00	0.00	0	0.00	0.00	5	0.00	0
	100	54.02	**275.2**	1	0.00	0.00	1	0.00	0.00	0	0.00	0.00	6	0.00	0

40	20	53.11	**486.6**	0	0.00	0.00	5	0.00	0.00	0	0.00	0.00	7	0.00	3
	40	60.02	**461.0**	0	0.00	0.00	5	0.00	0.00	0	0.00	0.00	10	0.00	3
	60	72.14	**416.4**	4	0.00	0.00	6	0.00	0.00	0	0.00	0.02	12	0.00	5
	80	79.09	**399.8**	8	0.00	0.00	6	0.00	0.00	1	0.05	0.25	12	0.00	5
	100	94.57	**377.0**	31	0.00	0.00	6	0.00	0.00	4	0.11	0.27	12	0.00	6

60	20	51.06	**581.6**	0	0.00	0.00	10	0.00	0.00	1	0.00	0.03	13	0.00	8
	40	58.64	**590.2**	1	0.00	0.00	10	0.00	0.00	1	0.12	0.41	16	0.00	37
	60	67.29	**560.0**	7	0.02	0.04	10	0.00	0.02	5	0.04	0.12	16	0.00	11
	80	77.25	**508.0**	47	0.00	0.08	10	0.00	0.02	6	0.24	0.39	16	0.20	18
	100	88.08	**514.8**	200	0.10	0.13	11	0.16	0.19	12	0.33	0.37	16	0.31	26

80	20	51.98	**676.6**	0	0.00	0.00	14	0.00	0.00	3	0.03	0.24	20	0.00	43
	40	59.14	**630.0**	3	0.00	0.00	15	0.00	0.00	9	0.02	0.03	21	0.00	69
	60	68.79	**606.4**	55	0.00	0.00	15	0.12	0.10	12	0.13	0.26	21	1.72	89
	80	72.67	**593.8**	220	0.33	0.09	15	0.13	0.17	14	0.29	0.29	21	0.10	60

100	20	47.58	**757.6**	103	0.00	0.00	19	0.00	0.01	9	0.03	0.11	24	0.00	175
	40	57.25	**701.8**	129	0.06	0.02	19	0.03	0.07	12	0.06	0.14	25	0.14	1
	60	67.98	**696.6**	148	0.08	0.02	19	0.01	0.03	13	0.17	0.43	25	0.00	148

150	20	47.82	**868.4**	2	0.00	0.03	27	0.05	0.06	16	0.12	0.21	36	0.02	420
	40	55.76	**834.8**	116	0.04	0.02	27	0.06	0.06	13	0.11	0.26	36	0.22	5
	60	61.56	**805.0**	463	2.44	0.39	27	2.09	0.21	18	2.10	0.60	37	1.91	630

200	20	47.86	**1009.0**	7	0.11	0.04	37	0.05	0.03	61	0.13	0.24	50	0.10	1456
	40	55.19	**984.2**	251	0.08	0.06	38	0.08	0.06	80	0.25	0.17	50	0.12	2106

**Table 7:** Mean CPU time (sec) comparison on the benchmarks.

	CPU MFlops	Benchmark set 1		Benchmark set 2		Benchmark set 3	
		Reported	Normalized	Reported	Normalized	Reported	Normalized
EMA-VP	~500	2.48	2.48	1.43	1.43	13.18	13.18
Beam-ACO	~2500	2.17	10.85	0.43	2.15	10.74	53.7
CA	~175	8.33	2.92	8.43	2.95	19.33	6.76
Heuristic	~200	5.79	2.32	—	—	197.18	78.87
